# Relation Between Exposure to Tobacco Smoke Assessed by Serum Cotinine Concentration and Questionnaire Method, and Serum Renalase Concentration—the Importance of the Coexistence of Arterial Hypertension and Other Cardiovascular Diseases

**DOI:** 10.1007/s12012-024-09868-z

**Published:** 2024-05-15

**Authors:** Aleksandra Żórawik, Wojciech Hajdusianek, Agnieszka Kusnerż, Iwona Markiewicz-Górka, Aleksandra Jaremków, Helena Martynowicz, Krystyna Pawlas, Grzegorz Mazur, Rafał Poręba, Paweł Gać

**Affiliations:** 1 Division of Environmental Health and Occupational Medicine, Department of Population Health, Wroclaw Medical University, Mikulicza-Radeckiego 7, PL 50-368 Wroclaw, Poland; 2 Department of Internal Medicine, Occupational Diseases, Hypertension and Clinical Oncology, Wroclaw Medical University, Borowska 213, PL 50-556 Wroclaw, Poland

**Keywords:** Tobacco smoke, Serum cotinine concentration, Serum renalase concentration, Arterial hypertension

## Abstract

Exposure to tobacco smoke (ETS) is one of the main risk factors for cardiovascular disease (CVD). Renalase is a protein that may play a role in the pathogenesis of CVD. The aim of the study was to assess the relationship between ETS and serum renalase concentration. A group of 109 patients was recruited for this study (49.7 ± 14.7 years). In accordance with the questionnaire, patients were divided into the following subgroups: subgroup A– declaring themselves active smokers (*n* = 36), subgroup B– declaring themselves non-smokers and exposed to environmental tobacco smoke (*n* = 35), subgroup C– declaring themselves non-smokers and not exposed to environmental tobacco smoke (*n* = 38). The same patients were divided based on cotinine concentration into the following subgroups: subgroup D– active smokers (*n* = 42), subgroup E– non-smokers exposed to environmental tobacco smoke (*n* = 66), and subgroup F– non-smokers not exposed to environmental tobacco smoke (*n* = 1). Serum cotinine concentration and serum renalase concentration were measured using ELISA tests. Serum renalase concentration was statistically significantly higher in subgroup C than in subgroups A and B and in subgroup E and F than in D. There was a negative correlation between serum cotinine concentration and serum renalase concentration (r = −0.41, p < 0.05). Regression analysis showed that higher BMI, higher diastolic blood pressure, coronary artery disease and higher serum cotinine concentration are independent risk factors of lower serum renalase concentration. The questionnaire method of assessing exposure to tobacco smoke was characterized by high sensitivity, but only moderate specificity, especially in terms of assessing environmental exposure to tobacco smoke. In summary, the study showed an independent relationship between exposure to tobacco smoke and lower serum renalase concentration.

## Introduction

Cardiovascular diseases are the leading cause of death and disability in the populations of developed countries. Hypertension is both a major risk factor for cardiovascular disease and an epidemiologically significant cardiovascular disease [1, 2]. Hypertension causes 9.4 million deaths each year and the number of people suffering from hypertension is increasing [1, 3, 4]. This is the result of global aging of societies and increasing exposure to modifiable risk factors, such as alcohol abuse, increased salt and calorie intake in the diet [1]. Coronary heart disease and stroke are the main consequences of hypertensive disease [5]. There is a relationship between socioeconomic status and cardiovascular diseases, including hypertension. These diseases are an unfavorable consequence of civilization development. However, the current studies indicate that the morbidity and mortality rates for hypertension in low-income countries are also increasing [1, 6–8].

Exposure to tobacco smoke is a serious health problem [9]. It includes not only active use of tobacco, which relationship with the development of negative changes in the cardiovascular system has been proven by numerous scientific studies, but also environmental exposure to tobacco smoke [10–16]. The research conducted so far indicates that environmental exposure to tobacco smoke may also have many serious negative consequences [12, 13, 17–21]. Second-hand smoke (SHS) and third-hand smoke (THS) are collectively referred to as environmental tobacco smoke (ETS) [20, 22]. WHO defines second-hand smoke as “the combination of smoke emitted from the burning end of a cigarette or other tobacco products and smoke exhaled by the smoker” [23]. Third-hand smoke is defined as “complex phenomenon resulting from residual tobacco smoke pollutants that adhere to the clothing and hair of smokers and to surfaces, furnishings, and dust in indoor environments” [22]. Current trends are noticeably reducing tobacco consumption in the last decade, but there are still over 1.3 billion people smoking cigarettes and over 8 million of them die every year due to tobacco smoke [16]. Exposure to tobacco smoke can be assessed based on the declarations of the examined persons. It is also possible to determine the concentration of nicotine metabolites in biological material, e.g., the cotinine concentration in blood, plasma, serum, urine or saliva [24].

Renalase is a relatively recently discovered flavoprotein that has been proposed to be an enzyme/hormone [25]. Although renalase was initially detected in the kidney, it can be found in a smaller amount in other organs and tissues such as the heart, small intestine, female and male gonads and skeletal muscles [26, 27]. This novel flavin adenine dinucleotide-dependent (FAD-dependent) amine oxidase has been documented to catabolize circulating catecholamines, causing a reduction in heart rate and blood pressure [26, 28, 29]. Later studies questioned the importance of catabolism of catecholamines by renalase [30, 31]. An earlier conclusion for renalase activity in catecholamine metabolism was made based on in vitro formation of hydrogen peroxide because of incubation of this enzyme with catecholamines. Based on subsequent analyzes, it was concluded that the formation of hydrogen peroxide was too slow to be attributed to the enzymatic activity of renalase [31]. However, there is still a scientific discussion in progress whether a renalase has a function in the metabolism of catecholamines. Currently, more and more scientific evidence seems to point the systemic importance of renalase as a compound with antioxidant potential [26, 28, 32, 33].

Renalase may therefore be a new pathogenetic link in the development of cardiovascular diseases. The search for relationships between risk factors for cardiovascular diseases, metabolic pathways of the genesis of these diseases and their clinical manifestations seems interesting.

The main aim of the study was to assess the relationship between exposure to tobacco smoke (ETS), i.e., active smoking and environmental exposure to tobacco smoke, and the serum renalase concentration. Additional goals included determining the significance of the occurrence of cardiovascular diseases, mainly arterial hypertension, for the analysed relationship; and the importance of the methodology for assessing exposure to tobacco smoke for the analysed relationship.

## Material and Methods

The study group was composed of patients of the Internal Medicine and Hypertension Clinic of the University Hospital in Wrocław (Poland). The following participants' inclusion criteria were used: adult patients hospitalized in clinic, consenting to participate in the study. The following were used as exclusion criteria: cancer, systemic diseases, chronic kidney disease, active inflammatory process, the ambiguity of smoking declaration, a history of one of the following conditions: cardiac or angio-surgery, stroke, myocardial infarction, and acute vascular incidents.

The required study group size was estimated using the sample size calculator. The following calculation criteria were assumed: estimated fraction size 50%, significance level 0.05, general population size 2,800,000, acceptable error 10%. Based on the above criteria, the required group size was calculated to be 97.

Finally, 109 patients participated in the study. A similar number of men (51.5%) and women (48.5%) participated in the study, with a mean age 49.7 ± 14.7 years and BMI 28.5 ± 5.3 kg/m^2^ (47.7% were overweight or obese). 37.6% of participants had arterial hypertension, 8.3% had type 2 diabetes, and 6.4% had coronary artery disease (without myocardial infarction). Table 1 describes the clinical characteristics of the study group.

**Table 1:** Characteristics of the study group (*n* = 109)

Age (years)^a^	49.7 ± 14.7/50.0 (24.0)
Gender^b^	
Men	51.5
Women	48.5
BMI (kg/m^2^)^a^	28.5 ± 5.3/28.8 (6.1)
Overweight^b^	20.2
Obesity^b^	27.5
Arterial hypertension^b^	37.6
Systolic blood pressure (mmHg)^a^	138.6 ± 20.5/135.0 (30.0)
Diastolic blood pressure (mmHg)^a^	89.2 ± 12.5/90.0 (15.0)
Mean blood pressure (mmHg)^a^	105.7 ± 14.4 / 105.0 (20.0)
Hypotensive drugs^b^	
ACE inhibitors	15.6
β-blockers	19.2
Diuretics	18.3
Calcium channel blockers	11.0
Angiotensin receptor blockers	9.2
Comorbidities^b^	
Type 2 diabetes	8.3
Coronary artery disease	6.4
Cotinine (ng/ml)^a^	16.4 ± 10.4/13.1 (7.5)
Renalase (ng/ml)^a^	189.7 ± 214.8/64.0 (318.6)

^a^arithmetic mean ± standard deviation/median (interquartile range)

^b^percentages

*ACE* angiotensin-converting enzyme, *BMI* body mass index

Subsequently, the study group were divided into subgroups based on different criteria. The first criterion was exposure to tobacco smoke based on participants’ declaration. The following subgroups were established: patients declaring active smoking (subgroup A), patients declaring non-smoking and exposure to environmental tobacco smoke (subgroup B) and patients declaring non-smoking and no exposure to environmental tobacco smoke (subgroup C). Environmental exposure to tobacco smoke was defined as staying at least 30 min a day in rooms where tobacco products are smoked or being in the immediate vicinity of people smoking tobacco products for at least 15 min a day or living with a person/people smoking cigarettes in the place of residence.

The second criterion was exposure to tobacco smoke assessed based on serum cotinine concentration. Based on this criterion, the study group was divided into the following subgroups: active smokers (subgroup D, serum cotinine concentration > 15 ng/ml), non-smokers exposed to ETS (subgroup E, serum cotinine concentration: 1–15 ng/ml) and non-smokers not exposed to environmental tobacco smoke (subgroup F, serum cotinine concentration < 1 ng/ml). The ranges of serum creatinine concentrations corresponding to types of exposure to tobacco smoke were adopted based on literature data [34–37].

The third criterion was a dichotomic division based on the presence of hypertension. Participants included to a group with arterial hypertension, were diagnosed according to the European Society of Cardiology guidelines. Arterial hypertension was diagnosed, when a mean of two measurements amounted to ≥ 140 mmHg in the case of systolic blood pressure and/or 90 mmHg in the case of diastolic blood pressure. In a situation when a participant declared administration of any hypotensive drugs, arterial hypertension was diagnosed independently of the measured values of arterial blood pressure.

The fourth criterion was self-reported exposure to tobacco smoke, but only in patients with hypertension. The fifth criterion was identical to the fourth one, but exposure to tobacco smoke was defined by serum cotinine concentration. The sixth and seventh criterion was analogical to the fourth and fifth criterion, respectively, but in the group without arterial hypertension. The divisions considering the criteria of type 2 diabetes and coronary artery disease were abandoned due to the low percentage of these diseases in the whole study group.

A tabular summary of the criteria for dividing the study group into subgroups is provided in Table 2.

**Table 2:** Serum renalase concentration in the study subgroups

Differentiation criterion	Subgroup	Renalase (ng/ml)^a^	*p* < 0.05
Exposure to tobacco smoke (based on the declaration)	A: patients declaring active smoking (*n* = 36)	81.4 ± 164.2/32.0 (19.4)	A vs. C B vs. C
	B: patients declaring non-smoking and exposure to environmental tobacco smoke (*n* = 35)	147.3 ± 178.2/82.4 (127.2)	
	C: patients declaring non-smoking and no exposure to environmental tobacco smoke (*n* = 38)	331.3 ± 215.3/356.8 (379.0)	
Exposure to tobacco smoke (based on serum cotinine concentrations)	D: active smokers (*n* = 42)	94.2 ± 165.7/35.1 (32.4)	D vs. E D vs. F
	E: non-smokers exposed to environmental tobacco smoke (*n* = 66)	246.7 ± 221.8/134.4 (348.9)	
	F: non-smokers not exposed to environmental tobacco smoke (*n* = 1)	441.5 ± 0.0/441.5 (0.0)	
Arterial hypertension (HTA)	HTA(+): patients with arterial hypertension (*n* = 41)	158.4 ± 207.7/56.0 (188.6)	ns
	HTA(−): patients without arterial hypertension (*n* = 68)	208.6 ± 218.4/91.4 (331.7)	
Exposure to tobacco smoke in patients with HTA (based on the declaration)	A/HTA(+): patients with arterial hypertension declaring active smoking (*n* = 17)	100.4 ± 205.7/28.1 (22.1)	A/HTA(+) vs. C/HTA(+) B/HTA(+) vs. C/HTA(+)
	B/HTA(+): patients with arterial hypertension declaring non-smoking and exposure to environmental tobacco smoke (*n* = 13)	93.4 ± 119.5/58.5 (42.8)	
	C/HTA(+): patients with arterial hypertension declaring non-smoking and no exposure to environmental tobacco smoke (*n* = 11)	324.7 ± 214.8/358.2 (368.9)	
Exposure to tobacco smoke in patients with HTA (based on serum cotinine levels)	D/HTA(+): active smokers with arterial hypertension (*n* = 21)	114.1 ± 202.4/31.6 (34.2)	ns
	E/HTA(+): non-smokers exposed to environmental tobacco smoke with arterial hypertension (*n* = 20)	204.8 ± 208.1/82.2 (333.2)	
	F/HTA(+): non-smokers not exposed to environmental tobacco smoke with arterial hypertension (*n* = 0)	–	
Exposure to tobacco smoke in patients without HTA (based on the declaration)	A/HTA(−): patients without arterial hypertension declaring active smoking (*n* = 19)	64.5 ± 119.2/36.0 (18.7)	A/HTA(−) vs. C/HTA(−) B/HTA(−) vs. C/HTA(−)
	B/HTA(−): patients without arterial hypertension declaring non-smoking and exposure to environmental tobacco smoke (*n* = 22)	179.2 ± 200.9/99.0 (157.3)	
	C/HTA(−): patients without arterial hypertension declaring non-smoking and no exposure to environmental tobacco smoke (*n* = 27)	334.1 ± 219.5/355.5 (406.5)	
Exposure to tobacco smoke in patients without HTA (based on serum cotinine levels)	D/HTA(−): active smokers without arterial hypertension (*n* = 21)	74.2 ± 120.3/36.8 (22.0)	D/HTA(−) vs. E/HTA(−) D/HTA(−) vs. F/HTA(−)
	E/HTA(−): non-smokers exposed to environmental tobacco smoke without arterial hypertension (*n* = 46)	265.0 ± 227.3/180.0 (373.0)	
	F/HTA(−): non-smokers not exposed to environmental tobacco smoke without arterial hypertension (*n* = 1)	441.5 ± 0.0/441.5 (0.0)	

^a^arithmetic mean ± standard deviation/median (interquartile range)

*HTA* arterial hypertension, *ns* non-statistically significant

The study methodology included anamnesis, cardiovascular diseases questionnaire, anthropometric measurements, blood pressure measurements, as well as serum cotinine and renalase concentrations.

To determine the serum cotinine concentration and the serum renalase concentration, approximately 10 cm^3^ of blood was collected from each subject from the arm vein. Blood was collected 12 h after the last meal into a tube containing EDTA, then the material was centrifuged at a speed of 10 000 revolutions/minute, and the collected serum was frozen and stored at -70 °C until the tests were performed.

Both serum renalase concentration and serum cotinine concentration in the study subgroups were measured using the enzyme-linked immunosorbent assay (ELISA). The determinations were performed strictly according to the test manufacturer's instructions.

Serum renalase determinations were performed using the E3109Hu kit ELISA (Bioassay Technology Laboratory, Shanghai, China). The renalase concentration was expressed as nanogram per milliliter (ng/ml). The reference range of the assay used was 1–400 ng/ml. According to the manufacturer, the sensitivity of the ELISA test used was 0.52 ng/ml. The coefficient of intra- and inter-assay variation was < 8% and < 10%.

Cotinine determinations were performed using the E2043Hu-96 T kit ELISA (Bioassay Technology Laboratory, Shanghai, China). The cotinine concentration was expressed as nanogram per milliliter (ng/ml). The reference range of the assay used was 0.5–80 ng/ml. According to the manufacturer, the sensitivity of the ELISA test used was 0.019 ng/ml. The coefficient of intra- and inter-assay variation was < 8% and < 10%.

Statistica 13 software for Windows was used for all statistical analyses. The arithmetic means, medians, standard deviations and interquartile ranges were calculated for quantitative variables. The normality of the distribution of quantitative variables has been verified. For quantitative variables with a normal distribution, the t-test or ANOVA (one-way parametric) analysis of variance with post-hoc tests were used for further statistical analysis. For quantitative variables with a non-normal distribution, the Mann–Whitney U test or a non-parametric equivalent of the analysis of variance, the Kruskal–Wallis ANOVA and post-hoc tests were used. The percentage distribution for categorical variables has been specified. Relationships between variables were assessed using correlation analysis, and univariate and multivariate regression analysis. In addition, the assessment of the sensitivity and specificity of the survey method of assessing exposure to tobacco smoke in relation to the determination of serum cotinine concentration as a reference method was made. The result at the level of p < 0.05 was considered significant.

## Results

Serum cotinine concentration in whole study group was 16.4 ± 10.4 / 13.1 (7.5) ng/ml [data presentation format: arithmetic mean ± standard deviation / median (interquartile range)] and serum renalase concentration was 189.7 ± 214.8 / 64.0 (318.6) ng/ml. Characteristics of the study group are shown in Table 1.

Comparative analysis of serum renalase concentrations in subgroups based on the criteria of exposure to tobacco smoke and hypertension showed statistically significant differences, Table 2. Patients declaring active smoking and patients declaring non-smoking and exposure to environmental tobacco smoke had significantly lower serum renalase concentrations than patients declaring non-smoking and no exposure to environmental tobacco smoke. However, when patients were analyzed not on basis of declaration, but in the context of cotinine concentration, active smokers were found to have significantly lower serum renalase concentrations than both non-smokers exposed to environmental tobacco smoke and non-smokers not exposed to environmental tobacco smoke. Hypertensive and non-hypertensive patients did not differ in serum renalase concentration. In hypertensive and non-hypertensive patients, similar differences in serum renalase concentrations between subgroups differing in exposure to tobacco smoke were found, as in the entire study group.

**Fig. 1:**
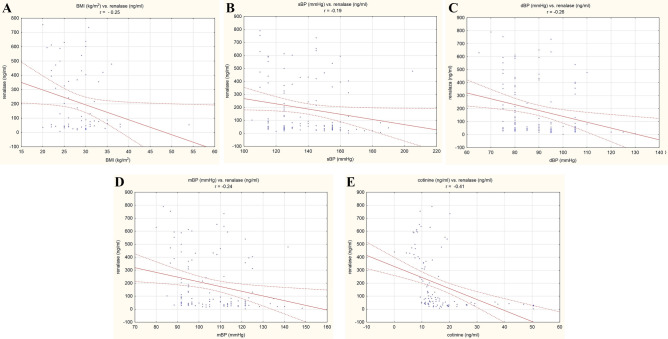
Correlations in the study group (*n* = 109). **A** BMI (kg/m^2^) vs. renalase (ng/ml). **B** systolic blood pressure (mmHg) vs. renalase (ng/ml). **C** diastolic blood pressure (mmHg) vs. renalase (ng/ml). **D** mean blood pressure (mmHg) vs. renalase (ng/ml). **E** cotinine (ng/ml) vs. renalase (ng/ml)

Serum renalase concentration was negatively corelated with BMI, systolic blood pressure, diastolic blood pressure, mean blood pressure and serum cotinine concentration, Fig. 1.

To analyze the relationship between serum renalase concentration and other factors the univariable and multivariable regression analysis was conducted. In the first step a univariate regression was performed to determine the variables associated with serum renalase concentration. It has been shown that there are relationships between BMI, systolic blood pressure, diastolic blood pressure, mean blood pressure, hypotensive drugs use, coronary artery disease and serum cotinine concentration with serum renalase concentration. Then in the second step, multivariable stepwise backward regression was performed, and it was shown that higher BMI, higher diastolic blood pressure, coronary artery disease and higher serum cotinine concentration are independent risk factors for lower serum renalase concentrations. The results of the regression analysis are presented in Table 3.

**Table 3:** Results of multivariable stepwise backward regression analysis in the study group (*n* = 109). Estimation for serum renalase concentration as the dependent variable

	Model for: renalase (ng/ml)						
	Univariate regression			Multivariable stepwise regression			
	Rc	SEM of Rc	*p*	Rc	SEM of Rc	*p*	*p* of the model
Age (years)	−1.130	1.406	0.423				0.012
BMI (kg/m^2^)	−10.441	4.933	0.038	−8.774	2.716	0.031	
Gender male^a^	53.126	41.044	0.198				
Arterial hypertension^a^	−50.286	42.401	0.238				
Systolic blood pressure (mmHg)	−2.007	0.993	0.046				
Diastolic blood pressure (mmHg)	−4.513	1.601	0.006	−3.243	0.893	0.014	
Mean blood pressure (mmHg)	−3.624	1.397	0.010				
Hypotensive drugs^a^	64.158	30.483	0.039				
Type 2 diabetes^a^	−89.275	74.620	0.234				
Coronary artery disease^a^	−157.454	78.951	0.040	−117.718	49.002	0.019	
Cotinine (ng/ml)	−8.501	1.814	0.011	−9.089	2.667	0.016	

^a^dichotomous variable, in which 1-yes, 0-no

The questionnaire method of assessing exposure to tobacco smoke was characterized by high sensitivity, but only moderate specificity, especially in terms of assessing environmental exposure to tobacco smoke. The sensitivity of the questionnaire assessment of exposure to tobacco smoke in relation to the assessment using serum cotinine concentration was 100% for both active smoking and environmental exposure. The specificity of the survey assessment of exposure to tobacco smoke in relation to the assessment using serum cotinine concentration was 85.7% for active smoking, but only 65.7% for environmental exposure. Several participants declared no exposure to environmental tobacco smoke but were found to be exposed in serum cotinine concentration assessment. The results of the sensitivity and specificity analysis of the test are presented in Table 4.

**Table 4:** Analysis of sensitivity and specificity of the declared exposure to ETS in relation to the reference method for the determination of serum cotinine concentration

A. Active smoking			
	Active smoking (based on serum cotinine concentration): NO	Active smoking (based on serum cotinine concentration): YES	Total
Active smoking (based on the declaration): NO	67	6	73
Active smoking (based on the declaration): YES	0	36	36
Total	67	42	109

Sensitivity		1.000	
Specificity		0.857	
Accuracy		0.945	
Positive likelihood ratio		7.000	
Negative likelihood ratio		0.000	
Positive predictive value		0.918	
Negative predictive value		1.000	
J Youden index		0.857	

B. Exposure to environmental tobacco smoke (ETS)			
	Exposure to ETS (based on serum cotinine concentration): NO	Exposure to ETS (based on serum cotinine concentration): YES	Total
Exposure to ETS (based on the declaration): NO	1	37	38
Exposure to ETS (based on the declaration): YES	0	71	71
Total	1	108	109

Sensitivity		1.000	
Specificity		0.657	
Accuracy		0.661	
Positive likelihood ratio		2.919	
Negative likelihood ratio		0.000	
Positive predictive value		0.026	
Negative predictive value		1.000	
J Youden index		0.657	

## Discussion

Renalase is a relatively recent discovery and still insufficiently investigated. The keyword “renalase” has only 255 Pubmed scores and 302 Scopus scores. In our study, we have documented that lower serum renalase concentration may be a consequence of cardiovascular risk factors as well as cardiovascular disease.

We obtained important results in relation to tobacco smoke exposure. In our study, we found that both active smoking and exposure to environmental tobacco smoke is related to lower serum renalase concentration. In the literature, we found only a few studies about the relationship between nicotine and renalase. In systemic review describing regulatory promoter and transcription factors of renalase gene, it was found that renalase promoter activity was augmented by nicotine [38]. This mentioned article is the only Pubmed and Scopus search result for the following search terms: ‘nicotine’, ‘renalase’, ‘tobacco’, ‘smoking’ in different combinations. Furthermore, one of research investigated the role of renalase in pancreatic cancer, high renalase (which promoter activity was increased by nicotine) was found to promote growth of pancreatic ductal adenocarcinoma (PDAC). High renalase concentration was associated with increase of PDAC mortality. Renalase concentration was inversely associated with metastatic melanoma [39].

Our study showed that blood pressure was statistically significantly related with serum renalase concentration. Systolic, diastolic, and mean arterial pressure were found to be negatively associated with serum renalase concentration. It has been documented that diastolic blood pressure is an independent risk factor for lower serum renalase concentration. The relationship between blood pressure and renalase has been documented in other studies to date. In research carried by Xianshu Li et al., aiming to determine renalase relation to pregnancy and preeclampsia, in a group of 384 Chinese participants, blood renalase concentration was negatively corelated with systolic and diastolic blood pressure. Study results were indicated that low blood renalase concentration could be a factor associated with increased risk of preeclampsia (PE) during pregnancy, and its gene polymorphism determined its blood concentration level and development of PE [40]. Similarly, in Polish study blood renalase concentration was lesser in patients with arterial hypertension [41].

We showed an independent relationship between coronary artery disease and lower serum renalase concentration. Few reports are available on the relationship between coronary artery disease and subsequent heart failure, and blood renalase concentration. Heart failure was found to be associated with blood renalase concentration decrease. In the study on rats, reduced renal blood perfusion (caused by heart failure) was found to be a possible cause of impaired renalase synthesis [42].

We found also that renalase concentration was independently inversely associated with BMI, but so far little has been published about this additional relationship. Similar results were obtained in a study conducted on 87 participants in which a negative correlation was observed between blood renalase concentration and BMI [41]. Similarly, Rybi-Szuminska et al. described a statistically significant negative correlation between urine renalase/creatinine ratio and BMI in a healthy pediatric population [43].

In addition, in our study, we examined whether the information declared by patients in the questionnaire about smoking corresponds to real exposure assessed by determining the concentration of cotinine in the serum. The questionnaire method of assessing exposure to tobacco smoke was characterized by high sensitivity, but only moderate specificity, especially in terms of assessing environmental exposure to tobacco smoke. This may be caused by lack of awareness about ETS exposure or neglecting it as non-important exposition. Similar problem was described by Benedetti et al. [44]. These authors emphasized that with the survey method of exposure assessment, respondents tend to give misleading answers about smoking, due to the currently growing awareness of the harmfulness of smoking, and thus the reluctance to admit to unhealthy habits, especially to medical personnel [44].

Our study has several important limitations that require discussion. The study group is relatively small. The minimum required size of the study group was estimated using a sample size calculator. A better solution would be to use multiple means test power analysis (one-way ANOVA). To confirm the results obtained and provide a more generalizable result, it would be necessary to perform the study again on a larger group of patients. Due to obtaining more accurate results and strengthening the level of evidence, it would be necessary to repeat the study with a larger number of participants, especially in the subgroup of those not actually exposed to ETS. Data from the survey method of assessing exposure to tobacco smoke should be interpreted with caution. Patients may tend to give answers that do not reflect the actual situation, as tobacco smoking may be perceived as an addiction they do not want to admit, especially to medical personnel. Moreover, it should be remembered that the term "exposure to tobacco smoke assessed by serum cotinine concentration" is a simplification. Cotinine measurements do not indicate sole tobacco smoke exposure, but exposure to any products containing nicotine. The study did not verify possible exposure to nicotine other than exposure to tobacco smoke. Other limitations of the study include the small number of patients with coronary artery disease and diabetes included in the study, the lack of data on the number of cigarette-years characterizing active smokers, the lack of determination of lipid profile and glycemia in the study group of patients, as well as information on the organ effects of hypertension assessed by imaging methods. In our opinion, the above limitations do not significantly reduce the value of the obtained results, but they may be a starting point for further research.

## Conclusion

There is an independent relationship between exposure to tobacco smoke and lower serum renalase levels, both for active smoking and for environmental exposure to tobacco smoke.

Higher BMI, higher diastolic blood pressure and coronary artery disease are risk factors for lower serum renalase levels independent of exposure to tobacco smoke.

The questionnaire method of assessing exposure to tobacco smoke was characterized by high sensitivity, but only moderate specificity, especially in terms of assessing environmental exposure to tobacco smoke.

## Data Availability

The data presented in this study are available upon request from the corresponding author. The data are not publicly available.
